# Evaluation of the chemical composition of nephrolithiasis using dual-energy CT in Southern Chinese gout patients

**DOI:** 10.1186/s12882-019-1441-8

**Published:** 2019-07-19

**Authors:** Zhao-Xia Li, Gen-Long Jiao, Shu-Min Zhou, Zhong Yuan Cheng, Shoaib Bashir, Yi Zhou

**Affiliations:** 1 Department of Rheumatology, The First Affiliated Hospital, Jinan University, No. 613 West Huangpu Ave, Tianhe District, Guangzhou, 510630 China; 2 Department of Orthopedics, The First Affiliated Hospital, Jinan University, NO.613 West Huangpu Ave,Tianhe District, Guangzhou, 510630 China; 3 Medical Imaging Center, The First Affiliated Hospital, Jinan University, No.613 West Huangpu Ave,Tianhe District, Guangzhou, 510630 China

**Keywords:** Primary gout, Dual-energy computed tomography, Nephrolithiasis, Risk factor, Biochemical abnormality

## Abstract

**Background:**

A study to evaluate the prevalence of uric acid (UA) nephrolithiasis with dual-energy CT (DECT) and explore the risk factors for kidney stones in primary gout patients.

**Methods:**

Eighty-four consecutive gout patients underwent urinary tract ultrasonography or DECT to confirm the existence of kidney stones. Urine and blood samples were also taken for laboratory analysis.

**Results:**

Forty-one subjects (48.8%) had nephrolithiasis diagnosed; 38 had a kidney stone. Thirty-two of the 38 patients underwent a DECT scan, and 27 patients had nephrolithiasis in DECT. Among them, 63.0% (17/27) and 14.8% (4/21) of the patients had pure UA and UA-based mixed stone, respectively, and 22.2% (6/27) had a non-UA stone. Those with nephrolithiasis suffered from more frequent acute attacks and had longer disease durations of gout. At least one urine biochemical abnormality was found in 81% of patients. Forty-four (55.0%) patients presented hypomagnesuria. Forty-three (51.8%) patients had low urine volume. Unduly acidic urine (UAU) was present in 36 patients (44.4%). Hyperuricosuria was only found in ten (12.2%) patients. In comparison to the non-lithiasic group, the lithiasic group was more likely to have a UAU. Binary logistic regression showed that female gender was a protective factor, while disease duration of gout and low urine pH were risk factors for nephrolithiasis.

**Conclusion:**

Our results indicated that nephrolithiasis, especially UA stones, were more common than previous reports in gout patients indicated, and that disease duration of gout, and low urine pH, were risk factors for nephrolithiasis.

## Background

Gout is a syndrome caused by over-produced UA, meaning that the latter deposits in different organs or tissues, causing various symptoms. The so-called curable way is urate-lowering therapy (ULT) [1]. In China, there are three urate-lowering drugs, which include inhibition of uric acid synthesis by xanthine oxidase inhibitors and enhancement of urate excretion by the uricosuric agent. The former include allopurinol and febuxostat, while benzbromarone is the only available uricosuric agent in China. The Han Chinese are susceptible to allopurinol hypersensitivity syndrome with a high HLA-B*58:01 allele carrier rate [2]. Febuxostat is very expensive and does not enter the health insurance catalogue, which limits its use in ordinary people. Benzbromarone, which is a well-defined urate-lowering drug, is the cheapest ULT agent and developing severe adverse reactions are not common in Chinese patients. The only drawback is that benzbromarone increases the risk of developing UA stones. According to the meta-analysis of observational studies, self-reported lifetime nephrolithiasis in people with gout was 24% [3]. However, not all kidney stones in gout patients consist of UA. If we can easily distinguish the composition of urinary stones, those with non-UA stones could benefit from benzbromarone, and the others, with UA stones, could be managed with conservative treatment instead of interventional procedures for stone removal or external shock-wave lithotripsy.

Nephrolithiasis has traditionally been evaluated using ultrasonography, plain film radiographic techniques, tomography or administration of intravenous contrast for excretory urography [4, 5], but neither of these methods discriminate stone composition before treatment. DECT is a new non-invasive technology that can differentiate UA from a non-UA kidney stone, which was proven in vitro experiments and in vivo by previous studies [6–9].

Our study aimed to explore the prevalence of UA kidney stones with DECT and evaluate the risk factors of nephrolithiasis in Southern Chinese gout patients.

## Methods

### Patients

Between January 2018 and November 2018, consecutive gout patients were recruited from the Department of Rheumatology of the 1st Affiliated Hospital of Jinan University. All patients fulfilled the 1977 ACR preliminary criteria for acute arthritis in primary gout [10]. Primary gout was diagnosed just after the exclusion of any other pathology or drug-associated cause of hyperuricemia. All patients who had a urinary tract infection or other conditions that could change urine traits were excluded from the study.

### Methods

#### Clinical evaluation

A researcher, who was blinded to the presence of nephrolithiasis, surveyed all related clinical and sociodemographic data from the subjects. All subjects were asked for their nephrolithiasis history, and kidney evaluation was undertaken via ultrasonography. The fasting blood samples of all the subjects were collected for biochemical tests.

#### Collection and analysis of urine sample

The first-morning samples of all the patients were collected for pH measurement and urine sediment analysis. All samples were processed within an hour. The 24 h urine samples were collected at 8:00 am, and the following 8:00 am. All participants were on a self-determined diet, and no individual instruction was given to restrict fluid or food intake during the sample collection period. No preservative was added to the 24 h urine sample, and it was stored at 4 °C. Volumes of 24 h urine samples were measured by a graduated cylinder. The urine samples were examined for calcium, sodium, UA, magnesium, phosphorus, potassium and creatinine.

#### DECT scan

**Fig. 1:**
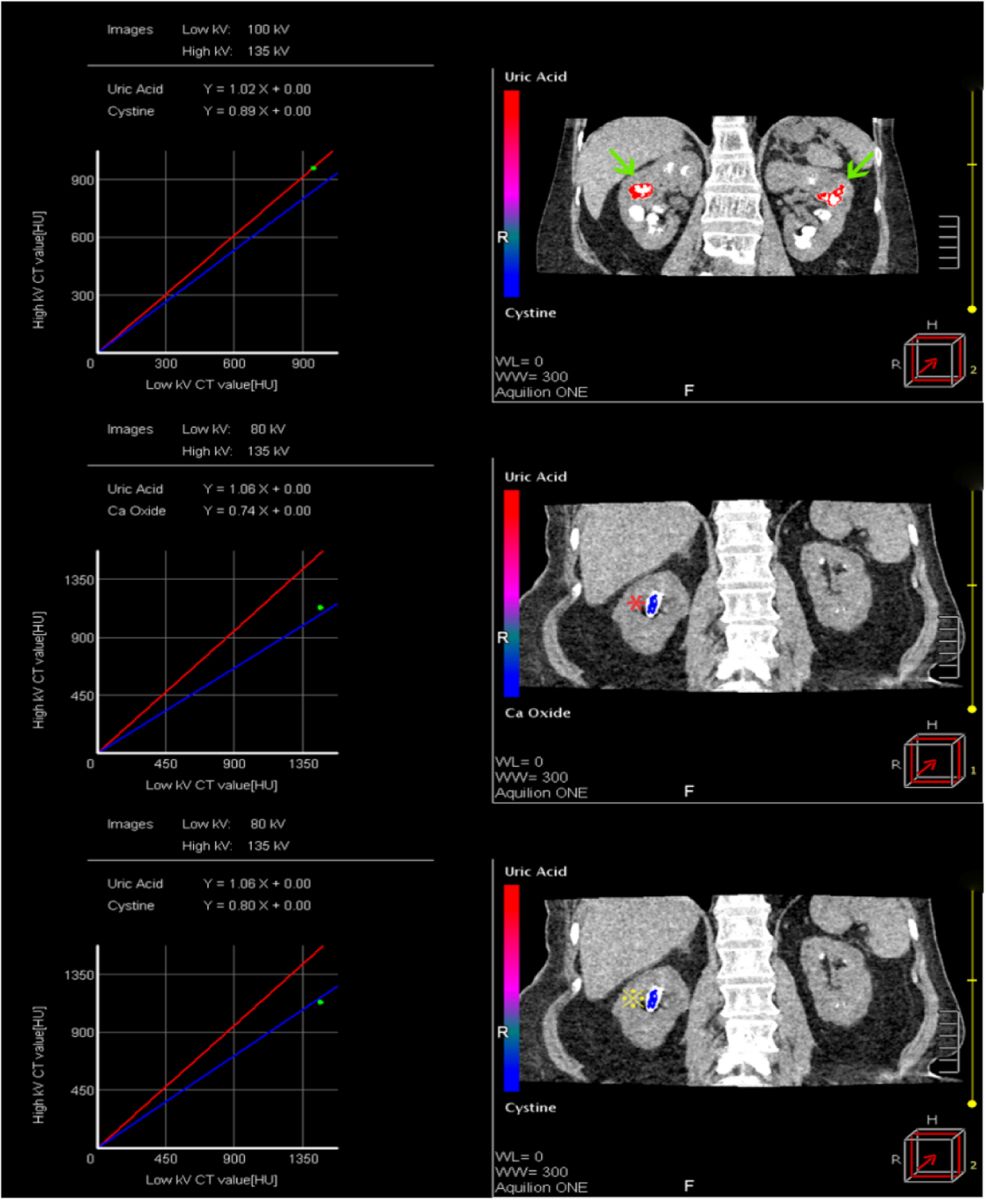
Shows the different compositions of kidney stones in the DECT scan. The green arrows (→) point to the uric acid stones in both kidneys. The red asterisk (*) shows the calcium stones and the yellow asterisk (※) points to the cystine stone

DECT was performed using a dual-energy helical scan mode with a 320-detector system (Aquilion ONE; Toshiba Medical Systems, Otawara, Japan). The combinations of tube current and tube voltage in scanning were 135Kv/80 mA and 100Kv/130 mA. The other scanning parameters are as follows: supine position, 160 mm range, 0.5 mm × 320 slices collimator, 512 × 512 matrices, 0.5 s per rotation and 1.5 s in total. The transverse datasets of both tubes were loaded into DE image view software (Toshiba Medical System, Tochigi, Japan) and reconstructed with the available software program - Stone Analysis (Toshiba Medical System, Tochigi, Japan). The region of interest was outlined as large as possible using a circular tool to cover each stone and axial, sagittal and coronal planes were taken as referred. Then, an image-based two-material decomposition algorithm of the datasets was subsequently performed to separate non-UA stone from UA stone, using soft tissue as the baseline (Fig. 1). Each DECT scan was analyzed by two CT radiologists. If they had a discrepancy in the cases, then they could seek advice from a senior radiologist and reach a consensus.. The radiologists were blinded to the clinical presentation of the patients.

### Definitions of nephrolithiasis and urinary biochemical abnormalities

Nephrolithiasis could be diagnosed if a previous surgical history for urolithiasis was acknowledged, or there was a history of passing a urinary stone, or positive findings were found on ultrasonography. Hypercalciuria was defined as urine calcium more than 300 mg/24 h for men and 220 mg/24 h for women. Hyperuricosuria was defined as urine uric acid more than 800 mg/24 h and 750 mg/24 h in men and women, respectively. Hypomagnesuria was defined as urine magnesium less than 60 mg/24 h. Hyperphosphaturia was defined as urine phosphate more than 768 mg/24 h in either sex. Low urine volume (LUV) was assumed when it was less than 2000 ml/24 h. Urine pH less than 5.5 was considered “UAU” [11].

### Statistical analysis

Comparison of baseline characteristics between lithiasic and non-lithiasic gout patients was performed using the Chi-square test for categorical variables and the Student’s t-test for continuous variables. Logistic regression was applied to examine the association of urolithiasis with independent variables, such as age, gender, the disease duration of gout, serum UA, serum creatinine, body mass index (BMI), urine pH and 24 h urine chemistry assay. Two-tailed *p*<0.05 was considered a significant difference. SPSS 20.0 software was utilized to conduct all statistical analyses.

## Result

A total of 84 patients (76 males and 8 females) were enrolled. Their demographic and clinical characteristics are shown in Table 1. None of the patients were taking any urate-lowering drug. None of the patients had ever been subject to interventional procedures or conservative treatment for stone removal.

**Table 1:** Demographic and clinical characteristics of non-lithiasic and lithiasic gout patients

Variable	Total (N=84)	Non-lithiasic (n=48)	Lithiasic (n=36)	*p*
Age, years ,mean	49.4±17.3	50.2 ± 2.8	48.4 ± 2.4	0.6398
Male sex, n(%)	76 (90.5%)	40 (83.3%)	36 (100%)	0.0093*
BMI, kg/m^2^, mean	26.2±3.6	26.7 ± 0.6	25.7 ± 0.5	0.2138
Disease duration	5.0±3.4	4.3 ± 0.5	6.0 ± 0.6	0.0249*
The frequency of gout flare, n(%)				0.0435*
Less than 3 times per year	22 (26.2%)	17 (35.4%)	5 (13.9%)	
≥3 times per year	62 (73.8%)	31 (64.6%)	31 (86.1%)	
Tophus, n(%)	26 (31.0%)	11 (22.9%)	15(41.7%)	0.0948
Number of involved joints,n(%)				0.0629
Only one joint	8 (9.5%)	6 (5.9%)	2 (5.5%)	
2-4 joints	29 (34.5%)	18 (37.5%)	11 (30.6%)	
Polyarthritis (≥5 joints)	47 (55.9%)	24 (50.0%)	23 (63.9%)	
Serum urate (umol/L)	533.6±156.3	551.3 ± 24.8	510.0 ± 22.0	0.2327
AP uric acid	65 (77.4%)	37 (77.1%)	28 (77.8%)	0.9400

*BMI* body mass index;

AP uric acid: the level of uric acid supersaturation(Serum urate >420umol/L)

*P* value stands for the Comparison between Non-lithiasic and Lithiasic group.

* indicate significant difference

Forty-one patients (48.8%) were diagnosed as nephrolithiasis, while only four patients had a history of renal colic. Ultrasonography examination was performed in all patients, while 38 currently had a kidney stone. Twenty-six patients had multiple calculi. The mean size of the calculi was 7 mm (range, 1.5–22.0 mm). Thirty-two of 38 patients had DECT scans, and the others refused. Comparing the DECT scans with ultrasonography findings, we found one patient with a positive finding on ultrasound but a negative DECT scan. Four patients were diagnosed with nephrolithiasis via ultrasonography, while their DECT scans showed renal calcification, rather than kidney stones. A total of 27 patients were found to have a kidney stone in the DECT scan. Among them, 63.0% (17/27) of the patients had pure UA, 14.8% (4/21) of the patients had UA-based mixed stone and 22.2%(6/27)had calcium oxalate or other compositions in the stone.

Demographic and clinical variables between the two groups are demonstrated in Table 1. Those with nephrolithiasis suffered a significantly higher frequency of acute gout attacks, and had longer disease duration of gout, while the prevalence of hypertension, diabetes, and hyperlipidemia between the two groups did not reach a significant difference.

Table 2 shows that urine UA, UA clearance and fractional excretion of UA were similar between the two groups. Urinary biochemical composition between the non-lithiasic and lithiasic groups was not statistically significant.

**Table 2:** 24 h urinary data in lithiasic patients and non-lithiasic patients

Variable	Non-lithiasic (*n* = 48)	Lithiasic (*n* = 36)	*p*
24h urine uric acid (mg)	3.2 ± 0.3	3.0 ± 0.2	0.5587
24h urine creatinine (mg)	11.6 ± 0.9	9.8 ± 0.7	0.1534
Ccr (ml/min)	98.7 ± 7.0	100.7 ± 8.7	0.8582
Cua (ml/min)	3.0 ± 0.3	3.1 ± 0.3	0.8260
FEua(%)	4.1 ± 0.6	3.5 ± 0.4	0.4961
24h urine Calcium(mg)	2.7 ± 0.4	3.2 ± 0.4	0.3283
24h urine Sodium(mg)	179.7 ± 14.8	161.0 ± 13.7	0.3726
24h urine Potassium(mg)	33.6 ± 2.5	34.6 ± 2.6	0.7860
24h urine Chloride(mg)	154.6 ± 14.1	153.8 ± 12.5	0.9703
24h urine Phoshate(mg)	12.4 ± 0.8	11.5 ± 1.1	0.4855
24h urine Magnesium(mg)	2.7 ± 0.2	2.4 ± 0.2	0.2912
Urine pH	6.0 ± 0.1	5.8 ± 0.1	0.1325
24h urine volume(L)	2.2± 0.1	2.1 ± 0.1	0.8688
No. of abnormality(%)			
hypercalciuria	2 (4.2%)	2 (5.6%)	0.7674
hyperuricosuiria	9 (18.8%)	1 (2.8%)	0.0253*
hypomagnesuria	24 (50.0%)	20 (44.4%)	0.6139
Hyperphosphaturia	1 (2.1%)	0	0.3836
LUV	24 (50.0%)	19 (52.8%)	0.8010
UAU	17 (47.2%)	19 (52.8%)	0.1116

*Ccr* creatinine clearance, *Cua* uric acid clearance, *FEua* fractional excretion of uric acid, *LUV* Low urine volume, *UAU* Unduly acidic urine

*indicate significant difference

As for biochemical abnormalities, 81% of patients had at least one abnormality. Forty-four (55%) patients presented hypomagnesuria. Forty-three (51.8%) had LUV. UAU was present in 36 patients (44.4%). Hyperuricosuria was found in only ten (12.2%) patients. Four (4.9%) patients were found hypercalciuric. Hyperphosphaturia was observed only in one patient. In contrast, lithiasic patients were more likely to have a UAU, while other abnormities were not significantly different between the two groups. Binary logistic regression showed that female gender was a protective factor, while the long disease duration and low urine pH was a risk factor for nephrolithiasis (Table 3).

**Table 3:** The risk factor of nephrolithiasis among primary gout patients

Variables	*B*	*S.E*	*Wald*	*Df*	*Sig.*	*Exp (B)*	*95% CI*	
							*Lower*	*Upper*
gout flare*	1.603	0.609	6.917	1	0.009	4.966	1.504	16.392
Constant	-1.504	0.553	7.404	1	0.007	0.222		

*S.E* Standard error, *95% CI* 95% confidence interval;

*using ‘less than 3 times per year’ as reference category

## Discussion

This research was the first in China to use DECT for distinguishing between uric acid and non-uric acid stones. The methodology is well established, and no proof was presented to demonstrate the possibility to make this distinction. No stone analyses were carried out. In our study, we found that nearly half of the primary gout patients had nephrolithiasis, while a few of them had symptoms, such as renal colic. Moreover, DECT results indicated that the majority of kidney stones in gout patients contained UA. The urine analysis only demonstrated that the prevalence of UAU was notably higher in the lithiasic group. Besides, we also found that female gender was a protective factor, while long disease duration and low urine pH were the risk factors for nephrolithiasis.

Nephrolithiasis is a common ailment not just in gout patients but also in ordinary people. The treatment of nephrolithiasis is partly based on the types of stones. Thus researchers have been trying to figure out an efficient non-invasive approach to predict stone composition before treatment for decades. Several in vitro and in vivo research studies have shown that DECT can discriminate UA from non-UA stone at low-dose radiation. In 2007, Primak et al. [12] had already testified that DECT could discriminate UA stone from other stone types in an anthropomorphic phantom model. The study showed that the DECT technique demonstrated 100% accuracy for the medium and large phantom. Even with the extra-large phantom (which was simulated in extra-large patient), DECT also had accuracy of over 93% and sensitivity of more than 88%. Some other studies also demonstrate that DECT could help differentiate between UA stone and others, especially in non-obese patients. Tomas et al. concluded that low-dose unenhanced dual-source DECT could help differentiate between calcified, UA and cystine calculi at a radiation dose similar to that of conventional intravenous pyelography. Based on the results mentioned above, we could see that DECT was an excellent tool to distinguish between UA and non-UA stone, which was also easy to access in Chinese medical units. According to our research, UA stone accounts for 77.8% of all kidney stones - higher than the previous report. Marchini et al. [13] reported that pure UA stone was present in 52.2% gout stone formers. The possible reason was that their stone samples were passed or retrieved surgically, but most of the kidney stones were asymptomatic, which may lead to an underestimated prevalence.

We wanted to know whether kidney stones were related to UA handling capacity. So we compared the indicators reflecting uric acid excretion function, but there was no significant difference between the two groups. We also found the uric acid excretion was not as high as we thought. The result was consistent with the previous study. Ma et al. [14] reported that most of their patients were “under-producers” of uric acid, only a few patients were referred to as overproducers or overexcretors. The cut-off in their study was 1000 mg/day as the lower limit for uric acid overproducers and patients with Cua ≥ 6 ml/min/1.73m^2^ was classified as overexcretors. In our study, only ten of our patients were overexcretors. Thus we speculated that the primary mechanism of gout was not overproduction but underexcretion of urate in Chinese patients. That was why the uricosuric agent was more effective and popular.

Sakhaee reviewed [15] that low urine pH, hyperuricosuria, and low urine volume were the main etiologic factors in UA stones. Partly consistent with their report, we found that nearly half of the participants had LUV and UAU. The lithiasic patients had a higher prevalence of UAU and lower average pH, but the latter did not reach statistical difference. Michael et al. [16] indicated that the formation of UA stone is primarily due to low urine pH, rather than excessive urinary concentration of UA. In the article, they depicted that at a urine pH of 5.5, even a modest concentration of UA level within the clinically normal range will lead to an undissociated UA level that far exceeds its solubility. Our results also illuminated that low urine pH was more critical in the formation of kidney stone than other factors. From this point, we could conclude that urine alkalizing agents are a crucial part of the treatment for preventing kidney stones in primary gout.

In recent years, the role of hypomagnesuria in kidney stones has received increasing attention. Hypomagnesuria was the most common biochemical abnormity in our study. Hussein et al. [17] reported that hypomagnesuria was present in 59.3% of Peninsular Malaysia urinary stone patients. Some authors also have observed hypomagnesuria as the most common metabolic alteration in patients with renal colic [18]. Some publications showed that a negative correlation was observed between erythrocyte magnesium and glycemic parameters in obese women, which suggested the influence of the mineral on the index of insulin resistance. The author also referred hypomagnesuria as a compensatory mechanism to keep the plasma magnesium within adequate levels [19]. Insulin resistance was the primary/underlying mechanism of gout, which may explain the hypomagnesuria in gout patients. The role of magnesium in inhibiting stone formation, and hence in the management of urolithiasis, remains to be verified by further studies.

Several pieces of evidence suggest that hypercalciuria is directly involved in the pathogenesis of stone formation. The prevalence of hypercalciuria has dramatically varied in different studies. Amaro et al. [20] reported that hypercalciuria was found in 50.8% of patients with urolithiasis, which was the most common urinary metabolic abnormity. However, it was present in 4.9% of the individuals in our study. Our result was in close agreement with the findings from the study in Peninsular Malaysia by Hussein et al. [17] and study in Thailand by Sriboonlue et al. [21], where both authors reported a low incidence of hypercalciuria (14.5, and 15%, respectively). The cause of hypercalciuria was multifactorial, which was hard to explain, but the region, diet culture, and climate may play a part.

Our results also demonstrated that the frequency of gout attacks was related to kidney stone formation. To our knowledge, increased frequency of acute gouty attacks means the more massive load of UA. The latter means the kidney must excrete more urate which increases the risk of kidney stone formation. Meanwhile, acute gouty arthritis has similar pathogenesis with crystal-induced renal damage, which are all related to NLRP3 inflammasome [22, 23]. We suspect that the mechanism of the deposition of urate in joints may be similar to the deposition of urate in the kidney.

However, our study has a few limitations. Firstly, patients in our department may have a more severe illness than primary care units, which caused a certain degree of selective bias. Secondly, a single-center study limits the number of subjects, which could be improved in the future by multi-center research studies. Thirdly, 24-h urine specimen collection may not be accurate enough, which would bring some error in the results.

## Conclusion

Our results indicated that nephrolithiasis was more common than the previous report in gout patients. UA stones were accounting for most of the nephrolithiasis, and its formation was primarily due to low urine pH. DECT scans can distinguish between UA stones and non-UA stones, which may help patients with UA stones benefit from conservative treatment and avoid interventional procedures. Besides, UAU was notably common in patients with nephrolithiasis, which indicates that urine alkalization may decrease the prevalence of nephrolithiasis in gout. Considering more acute gout attacks and longer disease duration were more common in patients with nephrolithiasis, thus tight control of uric acid to decrease acute flare could also be beneficial to nephrolithiasis.

## Data Availability

The datasets used and/or analyzed during the current study available from the corresponding author on reasonable request.

## Abbreviations

ACR: American College of Rheumatology
AP uric acid: The level of uric acid supersaturation
BMI: Body mass index
Ccr: Creatinine clearance
Cua: Uric acid clearance
DECT: Dual-energy computed tomography
FEua: Fractional excretion of uric acid
LUV: Low urine volume
UA: Uric acid
UAU: Unduly acidic urine
ULT: Urate-lowering therapy
